# Astrocytes Regulate Neuronal Network Burst Frequency Through NMDA Receptors in a Species- and Donor-Specific Manner

**DOI:** 10.1016/j.bpsgos.2024.100313

**Published:** 2024-04-03

**Authors:** Noora Räsänen, Jari Tiihonen, Marja Koskuvi, Šárka Lehtonen, Nelli Jalkanen, Nelli Karmila, Isabelle Weert, Olli Vaurio, Ilkka Ojansuu, Markku Lähteenvuo, Olli Pietiläinen, Jari Koistinaho

**Affiliations:** a Neuroscience Center, University of Helsinki, Helsinki, Finland; b Department of Clinical Neuroscience, Karolinska Institutet, and Center for Psychiatric Research, Stockholm City Council, Stockholm, Sweden; c AI Virtanen Institute for Molecular Sciences, University of Eastern Finland, Kuopio, Finland; d Department of Forensic Psychiatry, University of Eastern Finland, Niuvanniemi Hospital, Kuopio, Finland; e Helsinki Institute of Life Science, University of Helsinki, Helsinki, Finland; f Drug Research Program, Division of Pharmacology and Pharmacotherapy, University of Helsinki, FI, Helsinki, Finland

**Keywords:** Astrocytes, Induced pluripotent stem cells, Microelectrode array, Neurons, NMDA receptors, Schizophrenia

## Abstract

**Background:**

Development of synaptic activity is a key neuronal characteristic that relies largely on interactions between neurons and astrocytes. Although astrocytes have known roles in regulating synaptic function and malfunction, the use of human- or donor-specific astrocytes in disease models is still rare. Rodent astrocytes are routinely used to enhance neuronal activity in cell cultures, but less is known about how human astrocytes influence neuronal activity.

**Methods:**

We established human induced pluripotent stem cell–derived neuron-astrocyte cocultures and studied their functional development on microelectrode array. We used cell lines from 5 neurotypical control individuals and 3 pairs of monozygotic twins discordant for schizophrenia. A method combining NGN2 overexpression and dual SMAD inhibition was used for neuronal differentiation. The neurons were cocultured with human induced pluripotent stem cell–derived astrocytes differentiated from 6-month-old astrospheres or rat astrocytes.

**Results:**

We found that the human induced pluripotent stem cell–derived cocultures developed complex network bursting activity similar to neuronal cocultures with rat astrocytes. However, the effect of NMDA receptors on neuronal network burst frequency (NBF) differed between cocultures containing human or rat astrocytes. By using cocultures derived from patients with schizophrenia and unaffected individuals, we found lowered NBF in the affected cells. We continued by demonstrating how astrocytes from an unaffected individual rescued the lowered NBF in the affected neurons by increasing NMDA receptor activity.

**Conclusions:**

Our results indicate that astrocytes participate in the regulation of neuronal NBF through a mechanism that involves NMDA receptors. These findings shed light on the importance of using human and donor-specific astrocytes in disease modeling.

## Plain Language Summary

Nerve cell connections called synapses are formed in interaction with astrocytes, the main non-neuronal cell type of the brain. In vitro work commonly uses rodent astrocytes to enhance activity in human-derived neuronal cell cultures, but differences in using rodent versus human astrocytes are not well understood. We found that the electrical activity of nerve cell networks in cultures consisting of human cortical nerve cells and human astrocytes is altered when the astrocytes are from patients with schizophrenia, relative to neurotypical individuals. The effect of human astrocytes on these networks differed from rodent astrocytes, indicating the potential importance of a fully human culture system.

Synaptic maturation is a process that depends on surrounding astrocytes that actively regulate neuronal properties (1,2). To enhance the synaptic and functional maturation of neurons in vitro, neurons are often cocultured with astrocytes, typically derived from rodent brain (2,3). Despite the effectiveness of rodent astrocytes in supporting neuronal maturation, they differ from human astrocytes in terms of structural complexity, subtype diversity, developmental timeline, metabolic flexibility, response to inflammatory conditions, and many other features (4, 5, 6). This has raised a question about the extent to which rodent astrocytes can substitute for human glial cells and recapitulate features that are relevant for human neural functions. Furthermore, an increasing number of studies have shown that astrocytes have a role in the pathogenesis of developmental (1,7) and neurodegenerative disorders (8,9). This has demonstrated the need for fully human neuron-astrocyte cocultures for modeling development and disorders of the human brain.

## Methods and Materials

### Cell Lines

Human induced pluripotent stem cell (hiPSC) lines were derived from 5 neurotypical control (CTR) individuals and 3 pairs of monozygotic twins discordant for treatment-resistant schizophrenia. The patients with schizophrenia had a history of clozapine use (Table S1). The reprogramming and characterization of the hiPSC lines have been documented previously (10). All participants provided written informed consent, and the work was approved by the Ethics Committee of the Helsinki University Hospital District (license No. 262/EO/06).

### Neuronal and Astrocyte Differentiation

**Figure 1:**
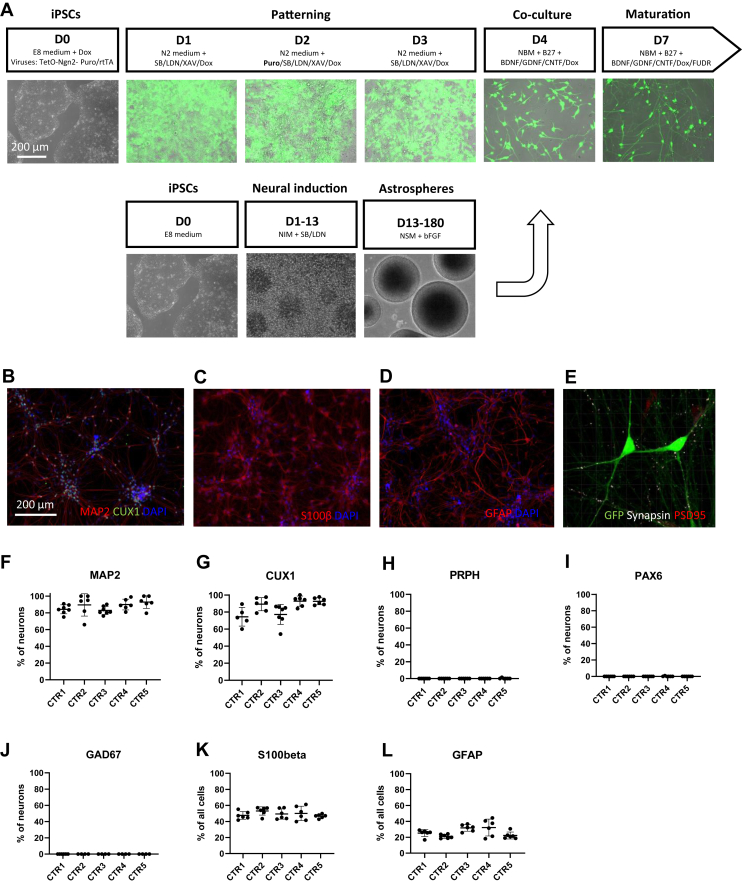
Characterization of hiPSC-derived neuron-astrocyte cocultures. **(A)** Differentiation of neurons and astrocytes. **(B, F, G)** Five-week-old neurons expressed neuronal marker MAP2 and superficial cortical layer marker CUX1. **(H–J)** The cultures did not stain for PRPH, PAX6, or GAD67, indicating the absence of peripheral neurons, differentiation-resistant PAX6-positive neural progenitor cells, and GABAergic neurons. **(C, D, K, L)** Approximately 50% of the cells in the cocultures were S100β-positive astrocytes, and 8%, 20%, and 30% of the cells expressed astroglial marker GFAP. **(E)** Neurons expressed colocalized synapsin and PSD95 proteins for juxtaposed pre- and postsynaptic puncta, respectively. This indicates the presence of structural excitatory synapses. (*n* = 5 cell lines; data were collected from 2–3 independent experiments). CTR, control; GABA, gamma-aminobutyric acid; GFP, green fluorescent protein; hiPSC, human induced pluripotent stem cell.

Neuronal differentiation was carried out by NGN2 overexpression coupled with dual SMAD and WNT inhibition, as previously described (2), to generate homogeneous populations of cortically patterned glutamatergic neurons (Figure 1A). The astrocyte differentiation and characterization has been described in our previous work (7,11). The full protocols are detailed in Supplemental Methods.

### Microelectrode Array Recordings

The electrophysiological activity was recorded with the Maestro Edge microelectrode array system using AxIS Navigator software and 24-well CytoView plates containing 16 electrodes (Axion Biosystems). The methods are further detailed in Supplemental Methods.

## Results

### Establishment of hiPSC-Derived Neuron-Astrocyte Cocultures

To study neuronal maturation in human neuron-astrocyte cocultures, we generated cultures of NGN2-expressing neurons and astrocytes from 5 neurotypical CTR individuals using previously published protocols (2,11). The neurons and astrocytes from the same individual were plated together after 4 days of neural induction (Figure 1A), and the maturation of the cocultures was continued until 5 weeks for immunocytochemical characterization. The neurons were labeled with GFP (green fluorescent protein) to separate them from astrocytes during the analysis (Figure S1A). After 5 weeks, 83% to 93% of the neurons expressed mature neuronal marker MAP2 (Figure 1B, F), and 71% to 95% of the neurons were positive for superficial layer cortical marker CUX1 (Figure 1B, G). Therefore, a great majority of the neurons had reached a mature neuronal identity and possessed features of excitatory superficial layer cortical neurons. Reassuringly, we observed minimal to no staining for contaminating cell types including peripheral neurons (PRPH), differentiation-resistant neural progenitor cells (PAX6), and GABAergic (gamma-aminobutyric acidergic) neurons (GAD67) (Figure 1H–J). Instead, we detected juxtaposed synapsin and PSD95 proteins along the neurites, which confirmed the presence of excitatory synapses in the cocultures (Figure 1E).

Finally, we used staining for canonical astrocyte markers to confirm the presence of the astrocytes in the cocultures. The astrocytes used in this study have been fully characterized before (7). After 5 weeks of maturation in cocultures, we detected S100β expression in 47% to 53% of the cells, whereas 21% to 32% of all cells expressed astroglial marker GFAP (Figure 1C, D, K, L). Given that we have previously shown that approximately 100% of these astrocytes express S100β, we concluded that the percentage of astrocytes in the cocultures remained close to the original 50%. In summary, we were able to produce homogeneous cocultures of NGN2-induced neurons and hiPSC-derived astrocytes with structural synapses.

### NGN2-Expressing Neurons Develop Synchronous Bursting Activity in Cocultures With hiPSC-Derived Astrocytes

After confirming robust neuronal maturation in cocultures with hiPSC-derived astrocytes, we set out to explore electrophysiological activity in these cultures. The functional development of NGN2-expressing neurons has previously been characterized in monocultures and in cocultures with rodent astrocytes (2,3). Typically, neurons in the mixed-species cocultures produce synchronous activity by 4 weeks of differentiation whereas the neurons in monocultures do not develop synchronous activity (2,3). To investigate whether NGN2-expressing neurons develop synchronous activity in cocultures with hiPSC-derived astrocytes, we prepared donor-specific cocultures of NGN2-expressing neurons and hiPSC-derived astrocytes from 5 cell lines for electrophysiological recordings. We compared the development of neuronal activity in the hiPSC-based cocultures with neuronal monocultures and mixed-species cocultures across 9 weeks of maturation on microelectrode array.

**Figure 2:**
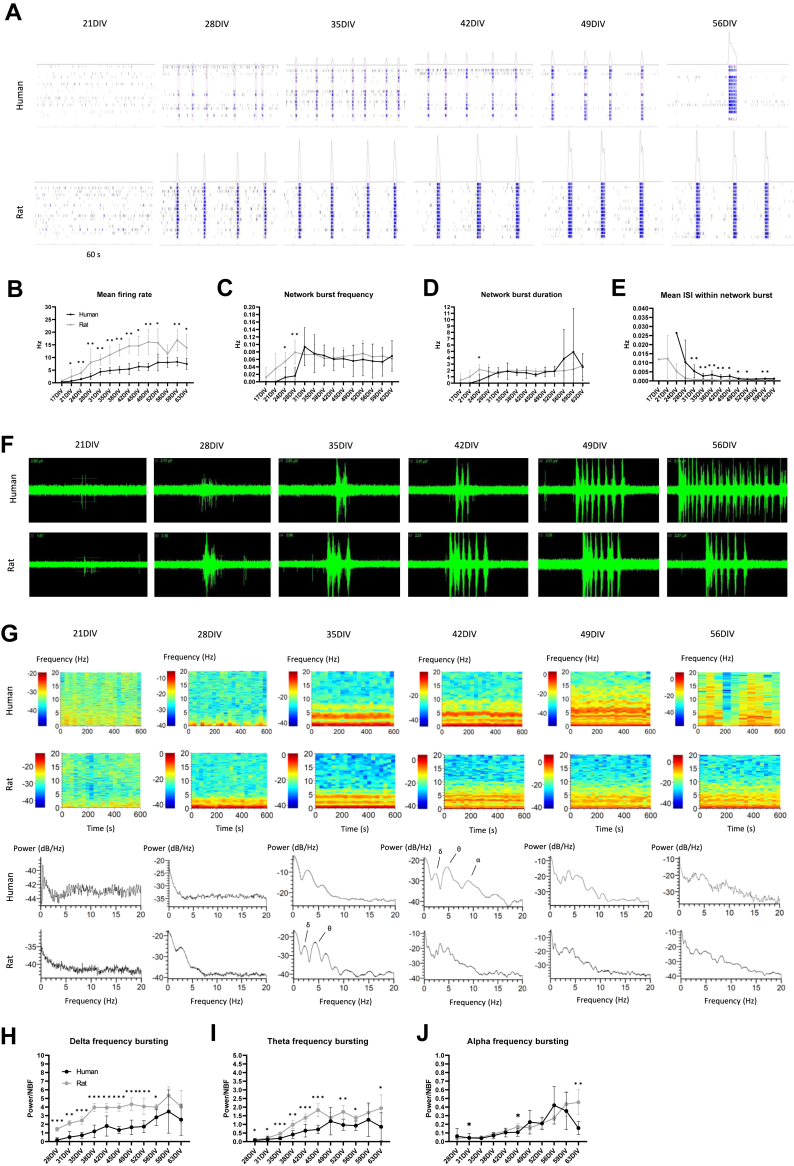
Timeline of the functional development of neurons in cocultures with hiPSC-derived astrocytes and rat astrocytes. **(A)** Development of network bursting activity in a typical cell line. **(B)** Neuronal mean firing rate increased across time in neuronal cocultures with human-derived or rat astrocytes. The mixed-species cocultures displayed higher mean firing rate throughout maturation. **(C)** Development of NB frequency in hiPSC-derived and mixed-species cocultures varied between cell lines. **(D)** The NB duration was stable until 49 DIV but exhibited high variability at later time points (>56 DIV) of hiPSC-derived cocultures. **(E)** The mean interspike interval within NB decreased gradually in both culture types. The hiPSC-derived cultures displayed higher mean interspike interval within NB across differentiation. (*n* = 5 cell lines; data were collected from 1 experiment). **(F)** Neurons developed high-frequency bursting activity within NBs in both hiPSC-derived and mixed-species cocultures. **(G)** The spectrograms and power spectral densities of the raw signal show increase in high-frequency bursting across time. By 5 to 6 weeks (35 DIV–42 DIV), distinct activity peaks were distinguishable at delta (δ), theta (θ), and alpha (α) frequencies. The heatmap values are presented in dB/Hz. **(H, I)** The mixed-species cultures displayed greater bursting activity at delta and theta frequencies than the hiPSC-derived cultures. **(J)** Alpha frequency bursting increased in both culture types at around 8 weeks of differentiation (56 DIV) (*n* = 8 samples from 1 cell line; data were collected from 1 experiment; Mann-Whitney *U* tests were used for the comparisons). ∗*p* < .05, ∗∗*p* < .01. DIV, days in vitro; hiPSC; human induced pluripotent stem cell; NB, network burst.

As previously reported, the neurons growing in monocultures did not develop synchronous activity (Figure S2) whereas the hiPSC-derived cocultures from all the cell lines developed synchronous network bursting (NB) activity by 5 weeks of differentiation (35 days in vitro [DIV]) (Figure 2A; Figure S3A). Overall, the emergence of the NBs was accompanied by an increase in mean firing rate (MFR) (Figure 2B; Figure S3A) that continued throughout neuronal maturation. In addition, all the lines presented sustained network burst frequency (NBF) and network burst duration (NBD) from 5 weeks onward (35 DIV) (Figure 2C, D; Figure S3A). The NBF and NBD had distinctive patterns between cell lines, and after 8 weeks of differentiation, a notable increase in the NBD was observed in 1 cell line (49–56 DIV) (Figure 2D; Figure S3A). The mean interspike interval (ISI) within the NB decreased gradually after 5 weeks of differentiation in all cell lines, indicating increasing spiking frequency within the NBs (35 DIV) (Figure 2E; Figure S3A). Taken together, the NGN2-expressing neurons generated sustained synchronous activity in cocultures with hiPSC-derived astrocytes after 5 weeks of differentiation.

Consistent with previous studies (2,3), the mixed-species cocultures developed synchronous NB activity by 4 weeks of differentiation (28 DIV) (Figure 2A; Figure S3B), suggesting that neurons in cocultures with human astrocytes developed slightly slower than those in cocultures with rat astrocytes. The mixed-species cocultures displayed higher MFR than the fully human cultures throughout differentiation (Figure 2B; Figure S3B), whereas the NBF and NBD converged to similar values in the mixed-species cultures, as was observed for fully human cultures (Figure 2C, D; Figure S3B). The mean ISI within the NB was lower in the mixed-species cultures than in the fully human cultures across maturation, indicating higher spiking frequency within the NB (Figure 2E; Figure S3B). Overall, the hiPSC-derived cocultures and mixed-species cocultures produced highly similar NB activity whereas the main differences between the models were found in the timing of the functional switch into NB activity and the overall MFR.

### NGN2-Expressing Neurons Develop Complex NB Activity in Cocultures With Human and Rat Astrocytes

After observing synchronous NB activity in the cocultures, we investigated the bursting patterns within the NB in more detail. In both hiPSC-derived and mixed-species cocultures, some neuronal lines developed complex bursting activity characterized by visible sub-bursts within the synchronous events (Figure 2F). Complex bursting that gives rise to oscillatory activity has been detected in long-term neuronal cultures such as brain organoids, but only after several months of maturation (12, 13, 14). To this end, we wanted to explore whether a similar phenomenon could occur in 2-dimensional cocultures and studied the development of bursting at high frequencies (>0.5 Hz) in one of the cell lines. First, we investigated power spectral densities and spectrograms of the activity at different time points and noticed increased power below 5 Hz at approximately 5 weeks of differentiation in human cocultures and approximately 4 weeks of differentiation in the mixed-species cocultures (35 DIV, 28 DIV) (Figure 2G). As we continued culturing the cells, the distinct bands diffused to a broader frequency spectrum (35–42 DIV) (Figure 2G). We measured the power of the activity at frequencies 0.5 to 4 Hz (delta), 4 to 8 Hz (theta), and 8 to 12 Hz (alpha) and noticed a steady increase in the activity at all frequencies over time in both culture types (Figure 2H–J). To summarize the findings, the hiPSC-derived neurons produced high-frequency bursting activity in cocultures with hiPSC-derived and rat astrocytes.

### Neuronal Response to NMDA Receptor Blocking Differs Between Human and Mixed-Species Cocultures

**Figure 3:**
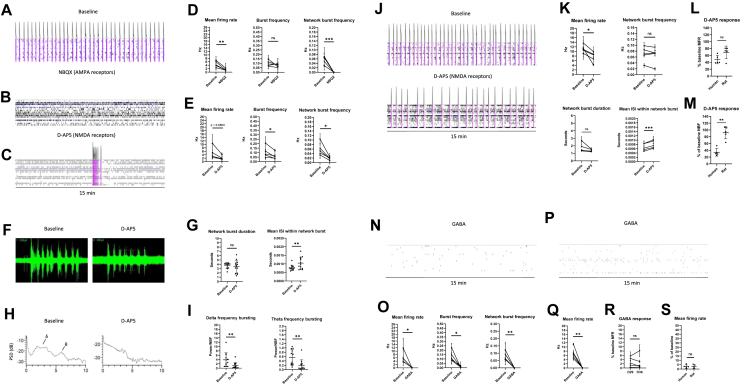
Functional characterization of neuron-astrocyte cocultures at 5 weeks of differentiation (35 DIV). **(A)** Raster plot image showing spiking, bursting, and NB activity recorded by each electrode at baseline. The hiPSC-derived cocultures generated regular synchronous activity by 5 weeks of differentiation (35 DIV). **(B)** AMPA receptors were blocked with 10 μM NBQX. **(C)** NMDA receptors were blocked with 25 μM D-AP5. **(D)** NBQX induced a significant decrease in mean firing rate and NBF but not burst frequency in the hiPSC-derived cultures. **(E)** D-AP5 induced a decrease in mean firing rate and a significant decrease in burst frequency and NBF (*n* = 5 cell lines; data were collected from 2–3 independent experiments). **(F)** Examples of bursts before and after D-AP5 treatment in hiPSC-derived cultures. **(G)** The NMDA receptor blockage did not affect NB duration but significantly increased the mean ISI within NBs. **(H)** PSDs showing changes in neuronal activity at different frequencies after the D-AP5 treatment. **(I)** NMDA receptor blockage significantly reduced bursting at delta and theta frequencies (*n* = 8–12 samples from 1 cell line, collected across 3 independent experiments). **(J)** Raster plot images showing synchronous activity in neuronal cocultures with rat astrocytes at 5 weeks of differentiation (35 DIV), before and after D-AP5 treatment. **(K)** The mean firing rate was significantly reduced after D-AP5 treatment whereas the NBF was not significantly affected by the treatment. The NB duration was not affected by the treatment whereas the mean ISI within NB was significantly increased after the D-AP5 treatment (*n* = 5 cell lines; data were collected from 1 experiment. Paired *t* tests were used for the statistical comparisons, and normal distribution of the data was verified with Kolmogorov-Smirnov test). **(L, M)** The reduction in NBF after D-AP5 treatment was significantly greater in hiPSC-derived cultures than in the mixed-species cultures at 35 DIV (*n* = 5 cell lines; data were collected from 1–3 independent experiments. Mann-Whitney *U* tests were used for the comparisons). **(N)** Response to 100 μM GABA in the hiPSC-derived cocultures. **(O)** GABA inhibited all bursting and virtually all spiking activity in the hiPSC-derived cultures**. (P, Q)** The mixed-species cocultures displayed an inhibitory response to GABA at 5 weeks of differentiation (*n* = 5 cell lines; data were collected from 2–3 independent experiments for iPSC-derived cultures and 1 experiment for mixed-species cultures. Paired *t* tests were used for the statistical comparisons; normal distribution of the data was verified with Kolmogorov-Smirnov tests). **(R)** The GABA response did not change between 4 and 5 weeks. **(S)** The GABA response did not differ between hiPSC-derived and mixed-species cocultures (*n* = 5 cell lines; data were collected from 1–3 independent experiments; Mann-Whitney *U* tests were used for the comparisons). ∗*p* < .05, ∗∗*p* < .01, ∗∗∗*p* < .001. DIV, days in vitro; GABA, gamma-aminobutyric acid; hiPSC, human induced pluripotent stem cell; ISI, interspike interval; NB, network burst; NBF, NB frequency; ns, nonsignificant; PSD, power spectral density.

After studying the development of neuronal activity across time, we wanted to understand how different neurotransmitter receptors contributed to the activity in the cocultures. Typically, hiPSC-derived neurons express robust AMPA receptor activity whereas NMDA and GABA receptor function are more rarely characterized despite their central role in brain disorders (2,4,10). To this end, we prepared hiPSC-derived cocultures from 5 donors and cultured the cells until 5 weeks of maturation. By this time, all the samples had developed synchronous activity. To first investigate AMPA receptor–mediated activity in the cells, we treated them with 10 μM AMPA receptor antagonist NBQX (Figure 3A, B). The treatment significantly reduced the MFR (*p* = .0057) and completely abolished NB activity in all the samples (Figure 3D). Similar abolishment of synchronous bursting has been observed previously in neuronal cocultures with rodent astrocytes (2).

Next, we investigated the role of NMDA receptors in neuronal activity by treating the cells with 25 μM of the NMDA receptor antagonist D-AP5 (Figure 3C). The treatment resulted in a nearly significant reduction in the neuronal MFR (*p* = .0503) and a significant reduction in burst frequency (*p* = .0362) (Figure 3E). The strongest effect was seen in NBF that was significantly reduced after the treatment (*p* = .0185) (Figure 3E). Previous studies have reported that NMDA receptors are necessary for the maintenance of high-frequency oscillatory activity in highly mature neuronal cultures (13). Therefore, we also studied the contribution of NMDA receptors to NB patterns in more detail using one of the cell lines. While there was no significant change in the NBD after D-AP5 treatment, the mean ISI within the NB was significantly increased, indicating reduced spiking within the NB (*p* = .0053) (Figure 3F, G). Next, we investigated whether a specific high-frequency bursting component within the NB was affected by the treatment and measured the effect of D-AP5 on bursting at delta and theta frequencies. As a result, we noticed a reduction in bursting at both frequencies (Figure 3H, I). To summarize the findings, NMDA receptors were identified as critical regulators of neuronal NBF, and they were found to enhance high-frequency bursting activity within the NB across the frequency spectrum.

To study whether the NMDA receptor–mediated activity differed in neuronal cocultures with human versus rat astrocytes, we blocked the NMDA receptors in the mixed-species cultures and compared the results with those obtained with the hiPSC-derived cocultures (Figure 3J). Given that the neurons matured at different rates in the fully human and mixed-species cocultures, we studied the NMDA receptor activity at both 4 and 5 weeks in the mixed-species cultures. At 4 weeks (28 DIV), the mixed-species cultures displayed a small reduction in MFR and NBF and an increase in the mean ISI within the NB after D-AP5 treatment, consistent with the human cultures (Figure S4). At 5 weeks, we noticed a reduction in MFR and an increase in mean ISI within the NB (Figure 3K) but no significant change in NBF (Figure 3K). We compared the reduction in MFR and NBF in the fully human and mixed-species cocultures at both time points and found that the magnitudes of change in response to D-AP5 was greater overall in the hiPSC-derived cultures for MFR and significantly greater for NBF (Figure 3L, M; Figure S4). Thus, the hiPSC-derived cultures displayed greater NMDA receptor activity that strongly affected neuronal NBF. Importantly, this result indicates that the effect of NMDA receptors on NBF was strongly and species-specifically regulated by astrocytes.

Given that astrocytes are known to express functional NMDA receptors, we sought to investigate the cell type–specific effects of neurons and astrocytes on the NMDA receptor function in the cocultures (15). In general, human astrocytes are larger than rodent astrocytes and can produce more NMDA receptors, resulting in stronger NMDA receptor activity (6). To first investigate whether the hiPSC-derived astrocytes were larger than the rat astrocytes, we measured their confluency in the cocultures. We found no differences in the area occupied by the astrocytes, which suggests that the human astrocytes were not larger than the rodent astrocytes (Figure S5A, B). We also investigated how the number of rat astrocytes in the mixed-species cocultures affected the NMDA receptor activity. We found that the astrocyte number did not significantly affect the neuronal response to D-AP5 (Figure S5A). To evaluate the number and distribution of NMDA receptors in the cultures, we measured the expression of GRIN1/GLUN1, which is a compulsory subunit present in all NMDA receptors. At the gene expression level, we found no evidence of higher GRIN1 expression in the hiPSC-derived cultures (Figure S5B). However, the immunocytochemical staining revealed that the NMDA receptors were strongly concentrated on neuronal somas and only weakly expressed by the astrocytes whereas no differences were found between the culture conditions (Figure S5C, D). Overall, these results suggest that the differences in the NMDA receptor function were caused by factors independent of NMDA receptor expression. Given that the NMDA receptors were only weakly expressed by the astrocytes, the NMDA receptor function was thought to originate from neurons (Figure S5D, E).

### NGN2-Expressing Neurons Display Inhibitory Response to GABA

After establishing a central role for the glutamate receptors in maintaining electrophysiological activity, we wanted to investigate the presence of functional GABA receptors in the cocultures. Although inhibitory neurotransmission is thought to be altered in many developmental and psychiatric disorders, the nature of the GABA response in hiPSC-derived models of the brain has rarely been characterized (10,14,16). First, we applied 100 μM GABA to hiPSC-derived cocultures at 5 weeks of differentiation (Figure 3N) and found that the neurons exhibited a strong inhibitory response to GABA. Altogether, GABA blocked 94% to 100% of the neuronal spiking activity and consequently all NB activity (Figure 3O). To find out whether the inhibitory response to GABA was a unique phenomenon for the hiPSC-derived cultures, we also investigated GABA responses in mixed-species cocultures after 4 and 5 weeks of maturation (Figure 3P). Already after 4 weeks, we observed a strong inhibitory GABA response (Figure 3P) that did not change between 4 and 5 weeks (Figure 3Q, R). The effect of GABA on the MFR did not differ between the fully human and mixed-species cultures (Figure 3S). Therefore, both human-based and mixed-species cocultures developed a fully inhibitory response to GABA by 4 to 5 weeks of differentiation.

### hiPSC-Derived Cocultures From Patients With Schizophrenia Show NB Alterations

Next, we investigated the suitability of the hiPSC-derived neuron-astrocyte cocultures for modeling cellular functions that underlie psychiatric disorders. We have previously performed transcriptomic profiling for neurons and astrocytes derived from monozygotic twins discordant for treatment-resistant schizophrenia. Using calcium signaling measurements, we have shown that neuronal NMDA receptor–mediated glutamate response differs between neurons from the affected twins (ATs) and neurons from their unaffected cotwins (UTs) in cocultures with astrocytes (7,10). To first investigate whether cocultures derived from these same cell lines displayed characteristic alterations in NB activity, we differentiated donor-matched neuron-astrocyte cocultures from hiPSCs derived from 3 monozygotic twin pairs discordant for treatment-resistant schizophrenia and 3 unrelated CTR individuals. After immunocytochemical staining and characterization of the cultures, we found no differences in the number of MAP2- or CUX1-positive neurons and detected no PAX6- or PRPH-positive cells in the cocultures. The number of S100β- and GFAP-expressing astrocytes was also similar across the conditions, consistent with our previous results (7) (Figure S6).

**Figure 4:**
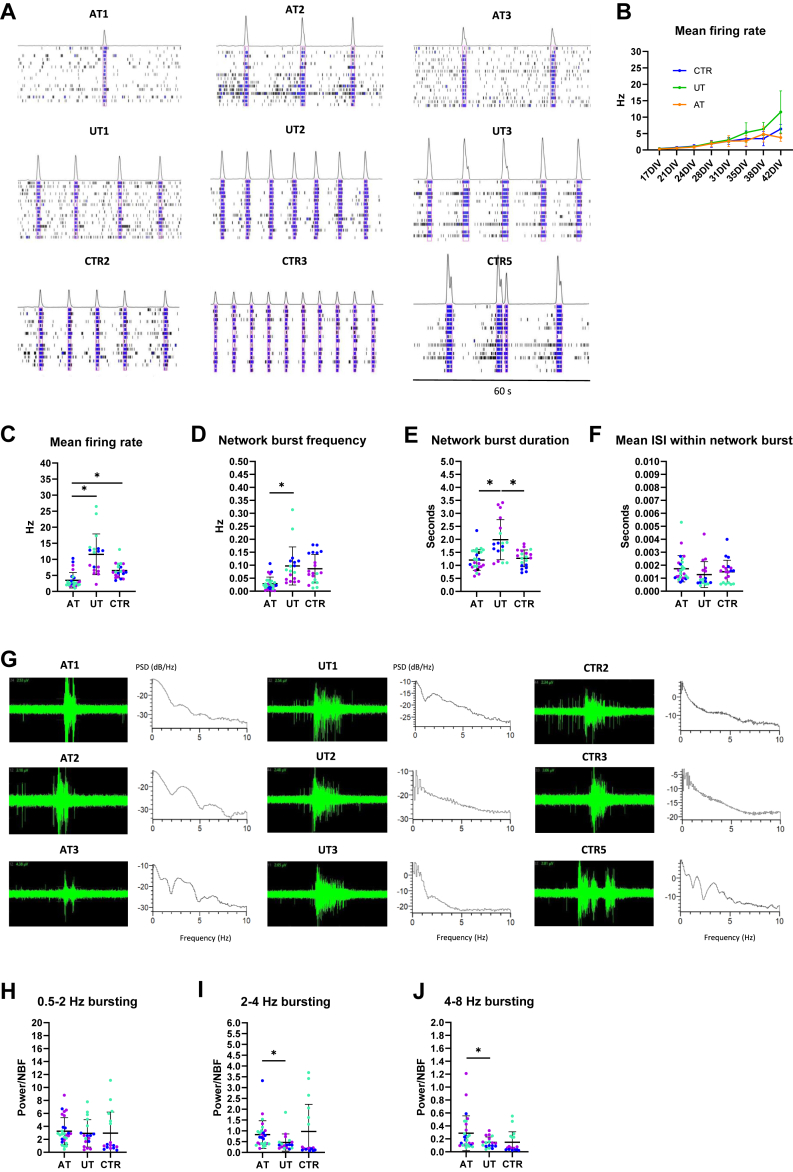
Characterization of neuronal activity in cocultures derived from monozygotic twins discordant for treatment-resistant schizophrenia and control individuals. **(A)** Raster plot images showing network bursting activity in cocultures derived from ATs, UTs, and CTR individuals at 42 DIV. **(B)** Development of mean firing rate in cultures derived from ATs, UTs, and CTR individuals. **(C)** Cocultures derived from UTs and CTR individuals displayed higher mean firing rate than cultures derived from the ATs at 42 DIV. **(D)** Cultures derived from UTs exhibited higher network burst frequency than cultures derived from ATs. **(E)** Cultures derived from UTs exhibited higher network burst duration than the cultures derived from ATs and CTRs. **(F)** The mean ISI within the network bursting did not differ between the cultures. **(G)** Raw signal showing burst shapes in cultures derived from ATs, UTs, and CTRs and PSDs showing the distribution of frequency components in the donor-specific cultures at 42 DIV. **(H–J)** Analysis of high-frequency bursting in AT, UT, and CTR cultures revealed differences between AT and UT cultures in 2 to 4 Hz bursting and 4 to 8 Hz bursting (*n* = 3 cell lines per group; data were collected from 1–2 independent experiments). *p* values were derived from analysis of variance using a general mixed linear regression model; the figures display uncorrected *p* values; ∗*p* < .05; the colors of the data points represent results from different cell lines. AT, affected twin; CTR, control; DIV, days in vitro; ISI, interspike interval; PSD, power spectral density; UT, unaffected twin.

Next, we prepared donor-matched cocultures on microelectrode array and monitored the development of neuronal activity until 42 DIV, when all the lines had developed robust NB activity (Figure 4A, B). At this stage, we noticed that both the UT and CTR cultures displayed higher MFR than the AT cultures (Figure 4B, C; Table S2; Figure S7). Overall, we found a prominent donor effect for MFR, with 38.77% of variance explained by the donor, whereas a similar 38.43% was explained by schizophrenia status. Belonging to the same twin pair did not contribute to the variance (Table S2). We further scrutinized the underlying factors of the differences in MFR and found that the UT cultures displayed higher NBF (*p* = .02389, *p*_adjusted_ = .16520) and NBD (*p* = .02236, *p*_adjusted_ = .16520) than the AT cultures (Figure 4D; Table S2). In addition, the UT cultures exhibited higher NBD than the CTR cultures (*p* = .03649, *p*_adjusted_ = .16520) (Figure 4E).

Although we found no difference between the groups in the mean ISI within the NB (Figure 4F), we did detect differences in the burst shapes between the AT and UT cultures. At this stage, the AT cultures displayed more distinct sub-bursts within the NB (Figure 4G). After quantifying neuronal bursting activity at high frequencies, we found stronger activity at 2 to 4 Hz and 4 to 8 Hz in the AT cultures than in the UT cultures (Figure 4J). In conclusion, using human-based cocultures, we were able to show differences in the activity of neurons from patients with schizophrenia and unaffected CTR individuals originating from NB patterns. Here, the difference in the activity between AT and UT cultures resulted from higher frequency and longer duration of NBs in the UT cultures. In addition, we found differences between the groups in high-frequency bursting patterns.

### The Role of Neuronal and Astrocyte Donor on NBF and NBD

**Figure 5:**
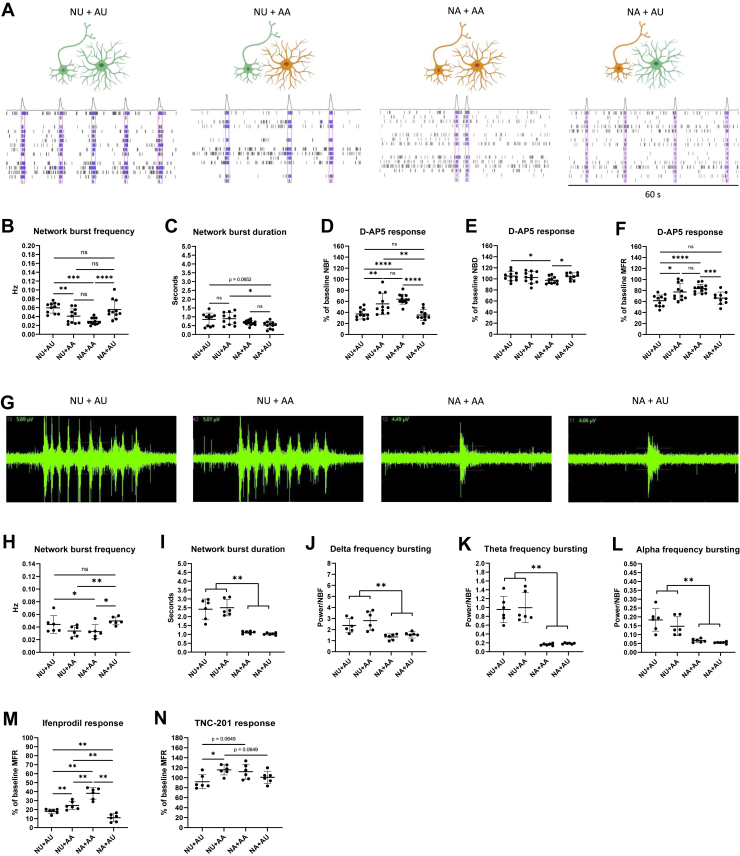
Characterization of neuronal activity in mixed-donor cocultures derived from 1 patient affected with schizophrenia and an unaffected twin. **(A)** Examples of network bursting activity in cultures with neurons and astrocytes from the UT (NU+AU), neurons from UT and astrocytes from AT (NU+AA), neurons and astrocytes from the AT (NA+AA), and neurons from the AT and astrocytes from the UT (NA+AU). **(B)** NU+AU cultures displayed significantly higher NBF than NA+AA cultures. NU+AA cultures displayed a significantly lower NBF than NU+AU cultures, and NA+AU displayed significantly higher NBF than NA+AA cultures. **(C)** The NB duration was slightly higher in cultures containing AU than in cultures containing AA. **(D)** The effect of D-AP5 on NBF was significantly stronger in NU+AU cultures than in NA+AA cultures. The NU+AA cultures displayed a significantly weaker D-AP5 effect of NBF than the NU+AU cultures. The effect of D-AP5 on NBF was similar to the level of NU+AU cultures when NAs were cultured with AU. **(E)** The effect of D-AP5 on NB duration was independent of neuron or astrocyte donor. **(F)** The effect of D-AP5 on MFR reflected the effect of D-AP5 on NBF (*n* = 10–12 samples collected across 2 independent experiments; Mann-Whitney *U* tests were used for the statistical comparisons) ∗∗∗∗*p* < .0001, ∗∗∗*p* < .001, ∗∗*p* < .01, ∗*p* < .05. **(G)** Raw signal showing bursts in mixed-donor cultures at 8 weeks of differentiation. **(H)** The NBF was significantly lower in cultures containing AA than in cultures containing AU. **(I)** NB duration was significantly elevated in cultures containing NU compared with cultures containing NA. **(J–L)** The power of delta, theta, and alpha frequency bursting was higher in cultures containing NU than in cultures containing NA. **(M)** The cultures containing AU displayed the highest response to the GLUN2B-specific blocker ifenprodil (10 μM). **(N)** GLUN2A-specific blocker TNC-201 (3 μM) slightly increased activity in cultures containing AA (*n* = 6 samples per condition; data were collected from 1 experiment; Mann-Whitney *U* tests were used for the statistical comparisons). ∗∗*p* < .01, ∗*p* < .05. AT, affected twin; MFR, mean firing rate; NBF, network burst frequency; ns, nonsignificant; UT, unaffected twin.

To investigate the role of neurons and astrocytes in regulating NBF and NBD, we selected neurons and astrocytes derived from one twin pair for more detailed characterization. We prepared mixed-donor cultures containing neurons and astrocytes from the UT (NU+AU), neurons from the UT and astrocytes from the AT (NU+AA), neurons and astrocytes from the AT (NA+AA), and neurons from the AT and astrocytes from the UT (NA+AU) (Figure 5A). We measured the neuronal activity at 6 weeks and compared the cultures in terms of NBF and NBD (Figure 5B, C). Interestingly, when the UT neurons were cultured with AT astrocytes, their NBF was significantly reduced (*p* = .0098). Moreover, when AT neurons were cultured with UT astrocytes, their NBF was significantly increased (*p* < .0001). This finding suggested that astrocytes critically influenced the neuronal NBF. When we measured the NBD in the different cultures, we found that the NBD was slightly higher in cultures containing UT neurons than in cultures containing AT neurons, suggesting that neurons, rather than astrocytes, regulated the NBD (Figure 5C). Taken together, we found that NBF and NBD were differentially influenced by neurons and astrocytes and that NBF was strongly affected by the astrocyte donor.

Given that we provided evidence earlier for the role of astrocytes in regulating NBF via NMDA receptors, we wanted to investigate whether the differences in NBF in cultures from affected and unaffected individuals were also mediated through NMDA receptors. We exposed the cultures to D-AP5 and measured the effect of the treatment on NBF. We found that the NBF in NU+AU cultures was reduced significantly more than in the NA+AA cultures (*p* < .0001) (Figure 5D), confirming that the UT cultures expressed stronger NMDA receptor–mediated activity. We also found that the NMDA receptor–mediated effect on NBF in AT neurons was fully rescued when cultured with UT astrocytes and significantly attenuated in UT neurons when cultured with AT astrocytes (*p* = .0066) (Figure 5D). Thus, the astrocytes had donor-specific, NMDA receptor–dependent effects on neuronal NBF. We also studied the effect of D-AP5 on NBD and found minor changes that were not explained by the neuron or astrocyte donor (Figure 5E). To test whether the phenomenon was consistent, we also prepared cocultures using cells from the other 2 discordant twin pairs and measured the effect of D-AP5 on MFR and NBF. In both twin pairs, the UT cultures displayed significantly higher D-AP5 response than the AT cultures, strengthening the idea that attenuated NMDA receptor activity was associated with schizophrenia (Figure S8).

Next, we wanted to investigate the effect of the neuron and astrocyte donor on the activity patterns within the NB. For this purpose, we continued culturing the cells for 8 weeks to see oscillatory activity within the network events (Figure 5G). At this stage, we observed significantly greater NBD and high-frequency bursting activity in cultures with UT neurons than in cultures with AT neurons (Figure 5H–L). Notably, the cultures with neurons from the same donor displayed highly similar NBD and high-frequency bursting patterns, suggesting that these features were exclusively regulated by neurons. Prolonged culturing of the cells also revealed that these differences were sustained across development (Figure S9).

Finally, we wanted to elucidate the role of NMDA receptors containing either GLUN2A or GLUN2B subunits in the electrophysiological activity of the cocultures. The 2 paralogs have different temporal expression profiles during brain development, with GRIN2B being highly expressed prenatally and GRIN2A becoming the dominant subtype after birth (17). We first used a GLUN2B-specific blocker ifenprodil (10 μM), to block the NMDA receptors in the mixed-donor cocultures (Figure 5M) and detected a strong response in all cultures. The most prominent response was observed in cultures containing UT astrocytes, suggesting that the differences in NMDA receptor function between the cultures were regulated by astrocytes through GLUN2B-containing receptors. Next, we blocked the GRIN2A-containing receptors with TCN-201 (3 μM) and observed a small increase in the MFR in cultures with AT astrocytes (Figure 5N). Based on this result, we concluded that GLUN2A-containing receptors played at most a minor role in regulating neuronal activity at this stage.

## Discussion

During the past few years, the importance of astrocytes in regulating neuronal activity has been increasingly recognized. Several hiPSC-based studies have demonstrated how the presence of astrocytes is required for the development of complex neuronal network-level activity in vitro (2,4,12,18). Astrocytes are also known to play a role in the pathogenesis of conditions such as schizophrenia and Alzheimer’s disease (1,7,8). Therefore, there is an increasing need for the use of donor-specific astrocytes in hiPSC-based models of the brain.

Here, we established fully human neuron-astrocyte cocultures derived from hiPSCs and detected development of synchronous NB activity in these cultures after 5 weeks of neuronal differentiation. We performed pharmacological characterization of the cocultures to study the contribution of different receptor types on the generation of NB activity. Although NMDA receptor dysfunction has been linked to many psychiatric disorders (10,19), the effect of these receptors on NB activity in hiPSC-derived neurons has not been characterized in detail. In our cultures, pharmacological blockage of NMDA receptors induced a strong reduction in NBF in contrast to the mixed-species cocultures. Similar, minor responses to NMDA receptor blockage have also been reported in other studies using NGN2-expressing neurons cultured with rodent astrocytes (2,20,21). This finding not only indicates that astrocytes influence the generation of synchronous activity in neuronal networks through NMDA receptors but also that this process is differently regulated by human and rodent astrocytes.

It is known that glutamate released from astrocytes can bind to extrasynaptic NMDA receptors in neurons, resulting in their synchronous excitation (22). Different chemicals released by the astrocytes are also known to specifically activate synaptic or extrasynaptic NMDA receptors (23). Thereby, the differences that we observed between the rat and hiPSC-derived astrocytes could originate from the release of different chemicals that activate specific receptors. Nevertheless, recapitulating the human-specific characteristics of NMDA receptor function in the neuron-astrocyte cocultures is critical for modeling disorders specific to the human brain.

Finally, we used the coculture model to investigate differences between brain cells derived from patients with schizophrenia and unaffected individuals. We were able to detect differences in the NB patterns between unaffected and affected cocultures at 6 weeks of differentiation. Previously, only one study had characterized neuronal NB patterns using hiPSC-derived neurons from patients with schizophrenia (24). This study also found evidence for reduced neuronal activity and NBF in the affected neurons after 6 weeks of differentiation. Along with our results, this finding indicates that astrocyte-driven NB activity may be critical for detecting disease-related functional alterations in neurons.

Intriguingly, we have consistently found differences between hiPSC-derived brain cells from identical twins that represent the same genetic background (7,10). In addition, other studies that have used hiPSCs from monozygotic twins discordant for schizophrenia have found differences between AT and UT cultures in neuronal activity and gene expression patterns (25, 26, 27, 28). These differences could be explained by de novo mutations carried by either one of the twins. We have earlier detected several nonoverlapping copy number variants between our twin lines (10). To further illuminate the disease mechanisms of schizophrenia in monozygotic twins, studies focusing on genetic and epigenetic differences between the twins would be needed.

## Supplementary Material

Key Resources Table
