# Supplementary material for: Astrocytes Regulate Neuronal Network Burst Frequency Through NMDA Receptors in a Species- and Donor-Specific Manner
Source: Biol Psychiatry Glob Open Sci. 2024 Apr 3;4(4):100313. doi: 10.1016/j.bpsgos.2024.100313 (PMC11067005; doi:10.1016/j.bpsgos.2024.100313)
Supplement: Supplemental Methods [file mmc2.pdf]

SUPPLEMENTARY INFORMATION

Astrocytes Regulate Neuronal Network Burst Frequency Through NMDA Receptors in Species and Donor-Specific Manner

Räsänen *et al.*

Supplemental methods

Neuronal differentiation

Neuronal differentiation was carried out by Ngn2 overexpression coupled with dual SMAD and WNT inhibition, as previously described (1), to generate homogenous populations of cortically patterned glutamatergic neurons (Figure 1A). The hiPSCs were infected overnight with 3 lentiviruses: Tet-O-NGN2-PURO, Tet-O-FUW-eGFP and FudeltaGW-rtTA (>10^9 IFU/ml, Alstem), using MOI = 10. The differentiation was initiated by adding 2 µg/ml Doxycycline hyclate (2431450, Biogems) in Essential 8 medium (A15169-01, Gibco). Doxycycline was used in the cell culture medium from this day onward. On day 1, N2 medium (DMEM/F-12 (21331-020, Gibco), 1:100 Glutamax (35050-038, Gibco), 1:100 N2 supplement (15410294, Gibco, 1:67 20% Glucose) was prepared and supplemented with 10 µM SB431542 (SB, S4317, Sigma), 100 nM LDN193189 (LDN, SML0559, Sigma), 2 µM XAV939 (2848932, Biogems) and 2 mg/ml Doxycycline. On day 2, the medium was changed to N2 medium supplemented with 1:2 of the day 1 supplements as well as 5 µg/ml puromycin (100552, MP biomedicals) to select for infected cells. On day 3, the cells were given the same media as on day 1. The final plating of the neurons was done on day 4. The cells were detached with Accutase (11599686, Gibco) for 5 min and centrifuged at 300 rcf for 4 min before counting. The cells were plated on wells coated with 50 µg/ml Poly-L-ornithine (Sigma, P3655) and 10 µg/ml laminin (Sigma, L2020). Neurons were plated 1:1 with astrocytes using 60 000 neurons/cm^2 for immunocytochemistry (ICC) and 60 000 neurons/well for microelectrode array (MEA; Supplemental Figure 1). Neurons were fed with Neurobasal medium (NBM, (21103-049, Gibco), 1:100 Glutamax, 1:200 MEM NEAA (11140-035, Gibco), 1:67 20% Glucose, 1:50 B27 without vitamin A (12587001, Gibco) supplemented with 10 ng/ml BDNF (450-02, PeproTech), 10 ng/ml GDNF (450-10, PeproTech) and 10 ng/ml CNTF (450-13, PeproTech) from day 4 onward. On days 7-8, proliferating cells were

eliminated from the cultures with 10 µM FUDR (4659, Tocris). Half of the medium was changed three times a week.

Astrocyte differentiation

The astrocyte differentiation was done as previously described (2,3). Briefly, neural induction was performed by culturing hiPSCs with 10 µM SB and 200 nM LDN. After 10 days of induction, NPCs arranged in rosettes were manually picked and transferred to ultra-low attachment plates, where the NPCs formed spheres. These spheres were cultured and expanded with bFGF (100-18B, Peprotech) for 6-9 months, after which they were dissociated and plated in co-cultures with neurons at 1:1 density. Astrocytes from different cell lines were cultured concurrently and represented the same age when plated. The astrocytes used in this study have been fully characterized before (3).

Rat primary astrocytes

Rat cortices were extracted from Wistar rat embryos at E17-18 as previously described (4). After initial cell preparation, the purified cortical cells were plated on T75 flasks with 20 ml DMEM High Glucose (ECB7501L, Euro Clone) supplemented with 10 % fetal bovine serum (10500-064, Gibco), 1 % L-Glutamine (BE17-605E, BioWhittaker) and 1 % Penicillin/Streptomycin (DE17-602E, BioWhittaker). After 7-8 days of culturing, the astrocytes reached confluency, and contaminating microglia and oligodendrocytes were removed by shaking the flask at 240 rpm for 6h as previously described (5). The cells were split using Trypsin (0.05%)-EDTA and the media was changed once every two weeks. The rat astrocytes were plated with hiPSC-derived neurons 1:1 using 60 000 astrocytes/well for MEA.

Immunocytochemistry

For immunocytochemical (ICC) staining, 5-week-old neurons were fixed with 4% formaldehyde for 20 min and washed twice with PBS. The cells were permeabilized with 0.25% Triton X-100 (T8787, Merck) in PBS for 1 hour. Unspecific binding sites were blocked using 5% normal goat serum (NGS, S26, Merck) for 1 hour. Primary antibodies against MAP2 chicken (1:500, Abcam, Ab92434), CUX1 mouse (1:500, Abcam, ab54583), VGLUT1 rabbit (1:300, Sigma, vo389-200), GAD67 mouse (1:500, Abcam, ab26116), PRPH rabbit (1:2000, Novus, NB300-137), PAX6 rabbit (1:500, Thermo, 42-6600), Synapsin Oyster 650 mouse (1:500, Synaptic Systems, 106011C5), PSD95 rabbit (1:500, Cell Signaling Technologies, 3450), GRIN1 rabbit (1:500, Cell Signaling Technologies, 5704), s100β rabbit (1:100, Abcam, 52642) and GFAP rabbit (1:500, DAKO, Z033429-2) were used. The primary antibody mixture was prepared in 5% NGS and incubated overnight at 4°C on a shaker. The cells were washed 3 x with PBS. Secondary antibodies including Goat anti-chicken Alexa Fluor 568 (A11041), Goat anti-rabbit Alexa Fluor 568 (A11011), Goat anti-mouse Alexa Fluor 633 (1:400, A21052, all from Thermo Fisher) were used. The secondary antibody mixture was prepared in PBS and incubated for 2 hours with the cells. Finally, the cells were washed 2 x with PBS and 1 x with DAPI (1:2000, Sigma) for 10 min. The samples were mounted using Fluoromount-G (00-4958-02, Thermo Fisher) and imaged using EVOS M5000 fluorescence microscope or Andor Dragonfly spinning disk confocal microscope (Nikon).

Image analysis

The image analysis for neuronal characterization was done with ImageJ (NIH). The neurons were labeled with GFP to separate them from astrocytes during the analysis, and the astrocytes were identified based on S100β expression. The images were thresholded using the default option to select for the cells expressing the target protein. The image calculator was used to co-localize the selected cells with a reference marker. DAPI was used as a reference for all cells and GFP was used

as a reference for neurons. The analyze particles feature was used to quantify the number of cells expressing the proteins of interest.

MEA recordings

The electrophysiological activity was recorded with Maestro Edge MEA system using AxIS Navigator software and 24-well CytoView plates containing 16 electrodes (Axion Biosystems). The recordings were performed at 37 °C temperature in a 5% CO_2 atmosphere. To start the measurements, the well plate was placed in the MEA system and the temperature and CO_2 were allowed to stabilize for 10 min. The baseline activity was measured for 10 min. For pharmacological tests, 10 µM NBQX (N138, Sigma), 25 µM D-AP5 (A8054, Sigma), 100 µM GABA (Sigma), 10 µM Ifenprodil (I2892, Sigma) and 3 µM TCN-201 (SML0416, Sigma) were used according to previous studies (1,6–8). The baseline activity was recorded for 10 min prior to treatment. After this, the pharmacological treatments were carried out by pipetting 5 µl of the compound into wells containing 500 µl of culture media. The plate was then placed back in the MEA device and incubated for 10 min before a 10 min recording was started.

MEA data analysis

The AxIS Navigator software was used for spike sorting during the recordings. The spike threshold was set to 5 x the standard deviation of the estimated noise. The burst detection was done using Neural Metric Tool (Axion biosystems). The minimum number of spikes per burst was set to 5 and the maximum inter-spike interval (ISI) within a burst was set to 100 ms. For NB detection, the Envelope algorithm was used due to its ability to merge repetitive sub-bursts within a network event into a single burst. A threshold factor value 2 and minimum inter-burst interval (IBI) 100 ms were selected for the analysis. The minimum number of electrodes in NB was set to 25 % and a burst inclusion value was set to 75%. The Envelope algorithm was not suitable for the analysis of NBQX-

treated samples that contained a great amount of non-synchronous bursting activity. Instead, we used Adapted algorithm that more efficiently separated the NBs from non-synchronous bursts. The minimum number of spikes per NB was set to 50 and the minimum number of electrodes in NB was set to 25 %. The NBs in the patient lines were analyzed using the max ISI algorithm that performed the best in terms of defining NB duration in samples with short bursts and bursting outside the NBs. The max ISI value was set to 50 ms and the minimum number of spikes per NB was set to 50.

NeuroExplorer (Plexon) software was used to analyze high frequency bursting activity in the samples. First, spike-sorted .spk files generated by the AxIS Navigator software were converted to rate histograms using a 0.025 s bin width in NeuroExplorer. Electrodes with fewer than 5 spikes per minute were removed from the files. Power spectral densities (PSD) were drawn for each electrode using Welch's windowing function. The same function was used for drawing the spectrograms. For visualization of spectrograms and PSDs, Log of PSD (dB) normalization was used, and for the analysis of high frequency bursting, raw PSD values were used. The power of the signal at frequencies 0.5-4 Hz, 4-8 Hz and 8-12 Hz was acquired from the PSDs for each electrode by averaging values within the defined frequency range. Finally, the values for each sample were acquired by averaging the values from the electrodes. The results for the power of high frequency bursting were presented as a ratio to the network burst frequency (NBF) in each well.

qRT-PCR

RNA was extracted from neuron-astrocyte co-cultures at 5 weeks using RNeasy Mini kit (74104, Qiagen) following the manufacturer's instructions. The extracted RNA was eluted in nuclease-free water. The cDNA conversion was performed with Maxima reverse transcriptase enzyme approach using Random hexamer primer (S0142, Fermentas), 10 mM dNTP (R0192, Fermentas), 40 U/µl Ribonuclease inhibitor (E00381, Fermentas) and Maxima reverse transcriptase (EPO742,

Fermentas). For the qPCR reaction, Maxima Probe qPCR Master Mix (K0261, Thermo Fisher Scientific) and primers for GRIN1 (Hs00287446_m1 and Rn01436030_m1, Thermo Fisher Scientific), GRIN2A (Hs00168219_m1, Thermo Fisher Scientific) and GRN2B (Hs01002013_m1, Thermo Fisher Scientific) were used. The gene expression was normalized to GAPDH (Hs99999905_m1, Thermo Fisher Scientific) or ACTB (Hs99999903_m1 and Rn00667869_m1, Thermo Fisher Scientific) using Q-gene program (9).

Statistical analysis

The statistical analysis was performed with GraphPad Prism 9.4.1 and RStudio 2022.12.0. Mann-Whitney U test was used to compare the differences between time points or co-cultures containing astrocytes from different species or donors. Paired t test was used for the investigation of pharmacological responses in neurons using GraphPad Prism. The normal distribution of the data was verified with Kolmogorov-Smirnov test. p-values for the comparison of cells from affected and unaffected individuals were derived from ANOVA using general mixed linear regression model in the lme package in R. The statistical tests for patient comparisons were corrected for multiple testing (28 tests) using Benjamin-Hochberg procedure implemented in GraphPad Prism.

Supplemental tables

Supplemental Table 1: *List of hiPSC lines used in this study.*

Cell line	Status	Age at biopsy	Sex	Medication
CTR1	control	44	Male	-
CTR2	control	49	Female	-
CTR3	control	64	Female	-
CTR4	control	63	Male	-
CTR5	control	50	Female	-
AT1	affected twin	47	Female	clozapine
AT2	affected twin	69	Female	previously clozapine, now sertindole and quetiapine
AT3	affected twin	45	Female	clozapine
UT1	unaffected twin	47	Female	-
UT2	unaffected twin	69	Female	-
UT3	unaffected twin	45	Female	-

Supplemental Table 2: *Statistical comparison of electrophysiological results for twins discordant for schizophrenia and unrelated controls at 42 DIV.*

**Variable**	**AT vs CTR**	**AT vs UT**	**UT vs CTR**	**AT vs Unaffected**	**Proportion of variance (ID/PairID/Status/Residual)**
MFR	-2.818 (1.060) p=0.04491 p_{adj} = 0.16520	-8.043 (2.887) p=0.02582 p_{adj} = 0.16520	5.297 (2.908) p=0.1034 p_{adj} = 0.24127	-6.709 (2.245) p=0.0472 p_{adj} = 0.16520	38.77/0/38.43/22.80
NBF	-0.05774 (0.03010) p=0.09087 p_{adj} = 0.24127	-0.07025 (0.02333) p=0.02389 p_{adj} = 0.16520	0.01283 (0.03924) p=0.7446 p_{adj} = 0.91694	-0.05302 (0.02999) p=0.09982 p_{adj} = 0.24127	50.26/0/15.93/33.81
NBD	-0.03953 (0.20347) p=0.8464 p_{adj} = 0.94797	-0.7559 (0.2574) p=0.02236 p_{adj} = 0.16520	0.7082 (0.2789) p=0.03649 p_{adj} = 0.16520	-0.3873 (0.2705) p = 0.1735 p_{adj} = 0.34700	26.31/0/30.21/43.48
mean ISI within NB	0.0001955 (0.0004065) p=0.6365 p_{adj} = 0.91694	0.0004576 (0.0003919) p=0.28 p_{adj} = 0.52267	-0.0002896 (0.0005882) p=0.6255 p_{adj} = 0.91694	0.0004465 (0.0004227) p = 0.3051 p_{adj} = 0.53393	32.47/0/0/67.53
0.5-2 Hz bursting	0.5457 (1.7296) p=0.7532 p_{adj} = 0.91694	0.4246 (1.1635) p=0.7165 p_{adj} = 0.91694	0.1237 (1.8729) p=0.9474 p_{adj} = 0.96090	-0.4496 (1.8486) p = 0.8081 p_{adj} = 0.94278	62.80/0/0/37.20
2-4 Hz bursting	-0.05501 (0.62841) p=0.9303 p_{adj} = 0.96090	0.3674 (0.1656) p=0.03211 p_{adj} = 0.16520	-0.4776 (0.6300) p=0.459 p_{adj} = 0.75600	0.1389 ((0.4361) p = 0.7506 p_{adj} = 0.91694	58.99/0/0/41.01
4-8 Hz bursting	0.14544 (0.09605) p=0.1604 p_{adj} = 0.34548	0.1475 (0.06163) p=0.04062 p_{adj} = 0.16520	-0.004097 (0.083547) p=0.9609 p_{adj} = 0.96090	0.13710 (0.06633) p = 0.05893 p_{adj} = 0.18334	21.72/0/5.3/72.97

Supplemental Figures

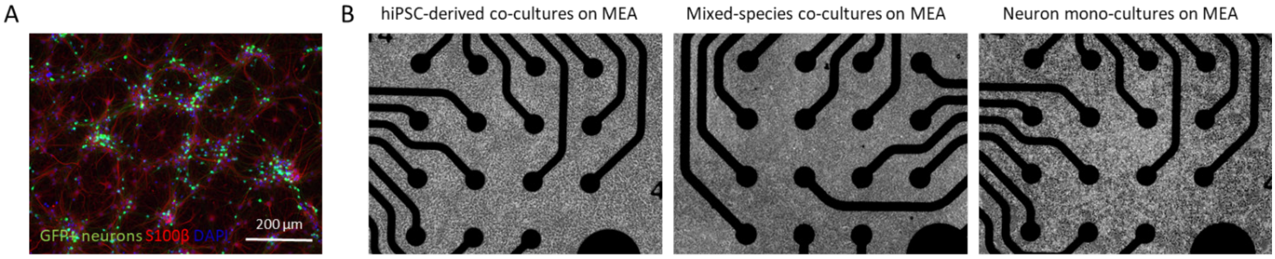

Supplemental Figure 1: hiPSC-derived neurons and astrocytes in co-cultures. A. Neurons were labeled with GFP for ICC characterization. B. Co-cultures and neuronal mono-cultures plated on MEA.

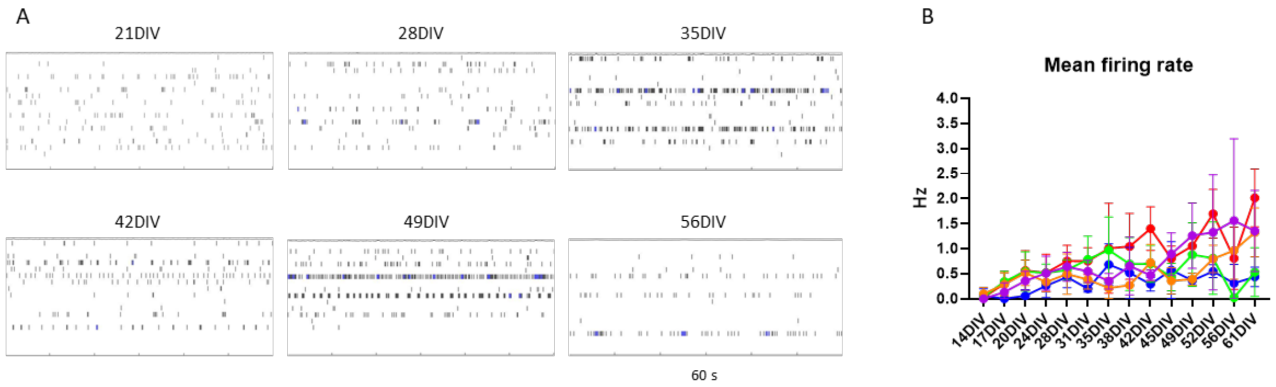

Supplemental Figure 2: Functional characterization on neuronal mono-cultures on MEA. A. Raster plot images showing neuronal spiking and bursting activity. B. MFR across time measured from 5 cell lines.

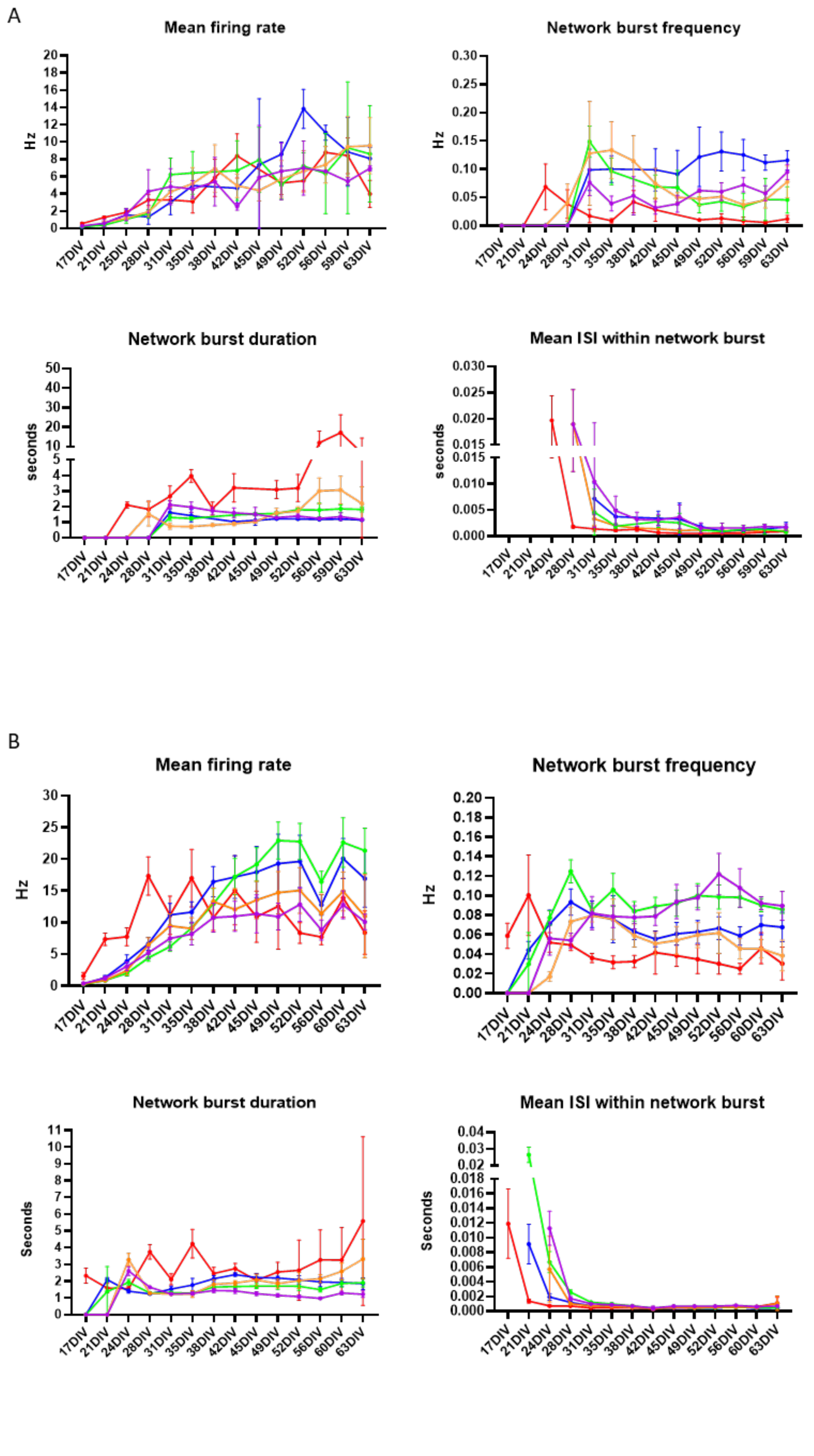

Supplemental Figure 3: Timeline of the functional development of neurons with hiPSC-derived astrocytes (A) and rat astrocytes (B).

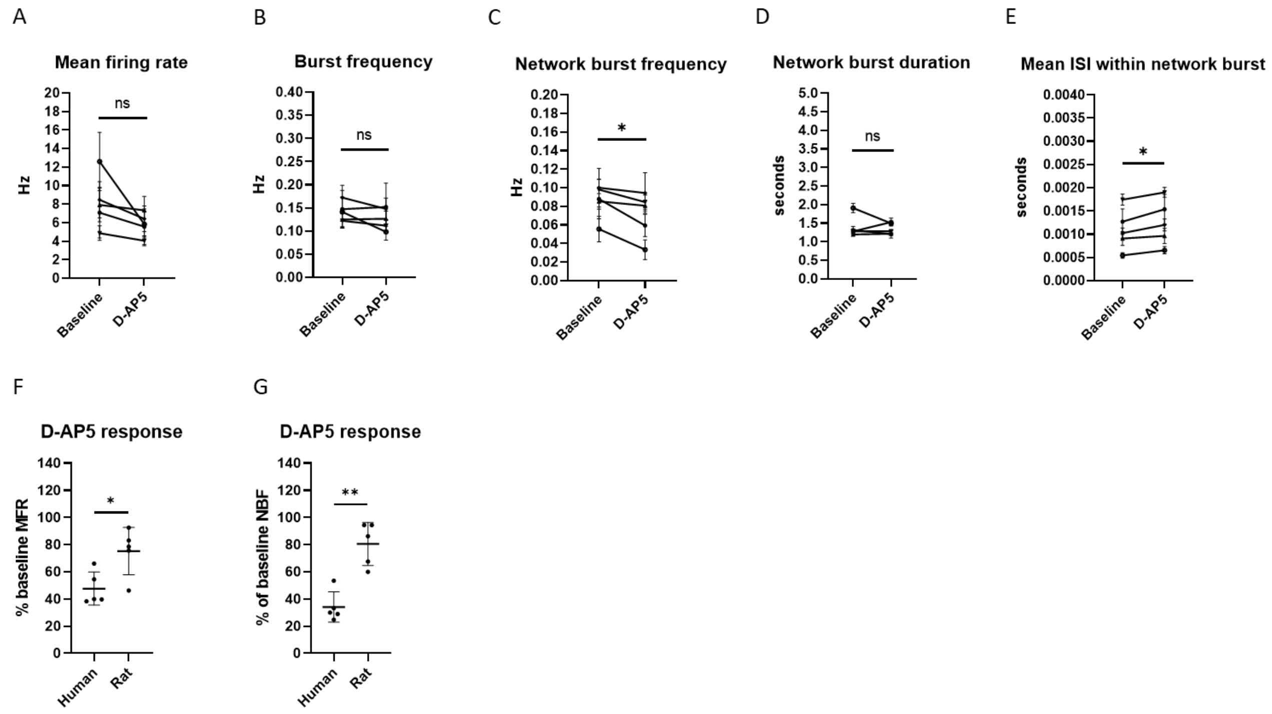

Supplemental Figure 4: Characterization of neuronal D-AP5 response in co-cultures with rat astrocytes at 4 weeks of differentiation. A-E. The NMDA receptor blockage had a significant effect on NBF and mean ISI within NB in the mixed-species cultures. F-G. The hiPSC-derived cultures displayed a stronger response to D-AP5 than the mixed-species co-cultures. (n = 5 cell lines, data was collected from 1 experiment. For A-E: Paired t test was used for the statistical comparisons, normal distribution of the data was verified with Kolmogorov-Smirnov test. For F-G: Mann-Whitney U test was used for the statistical comparisons. * signifies $p < 0.05$, ** signifies $p < 0.01$, ns = non-significant)

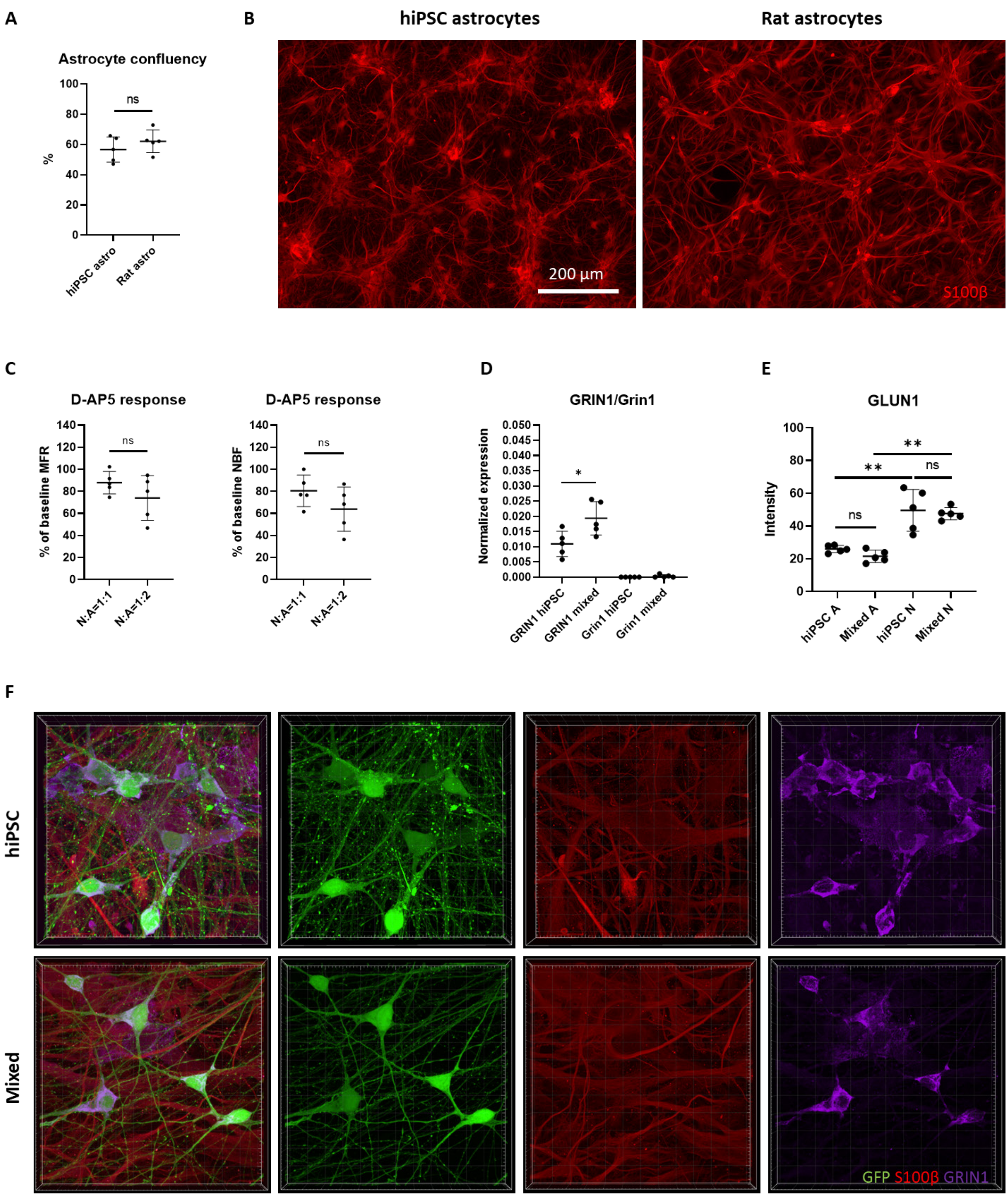

Supplemental Figure 5: Characterization of NMDA receptors in neuron-astrocyte co-cultures at 5 weeks of differentiation. A-B. The astrocyte confluency did not differ between hiPSC-derived and mixed-species co-cultures. C. The number of astrocytes did not significantly affect the NMDA receptor activity in mixed-species co-cultures. D. qPCR quantification of human-specific GRIN1 and

rat-specific Grin1 subunits in hiPSC-derived and mixed-species co-cultures. E-F. ICC quantification of GRIN1 subunit expression in hiPSC-derived and mixed-species co-cultures revealed higher NMDA receptor expression in neurons compared to astrocytes in both culture conditions. (n=5 cell lines, data was collected from 1 experiment, Mann-Whitney U test was used for the statistical comparisons, * signifies $p < 0.05$, ** signifies $p < 0.01$, ns = non-significant)

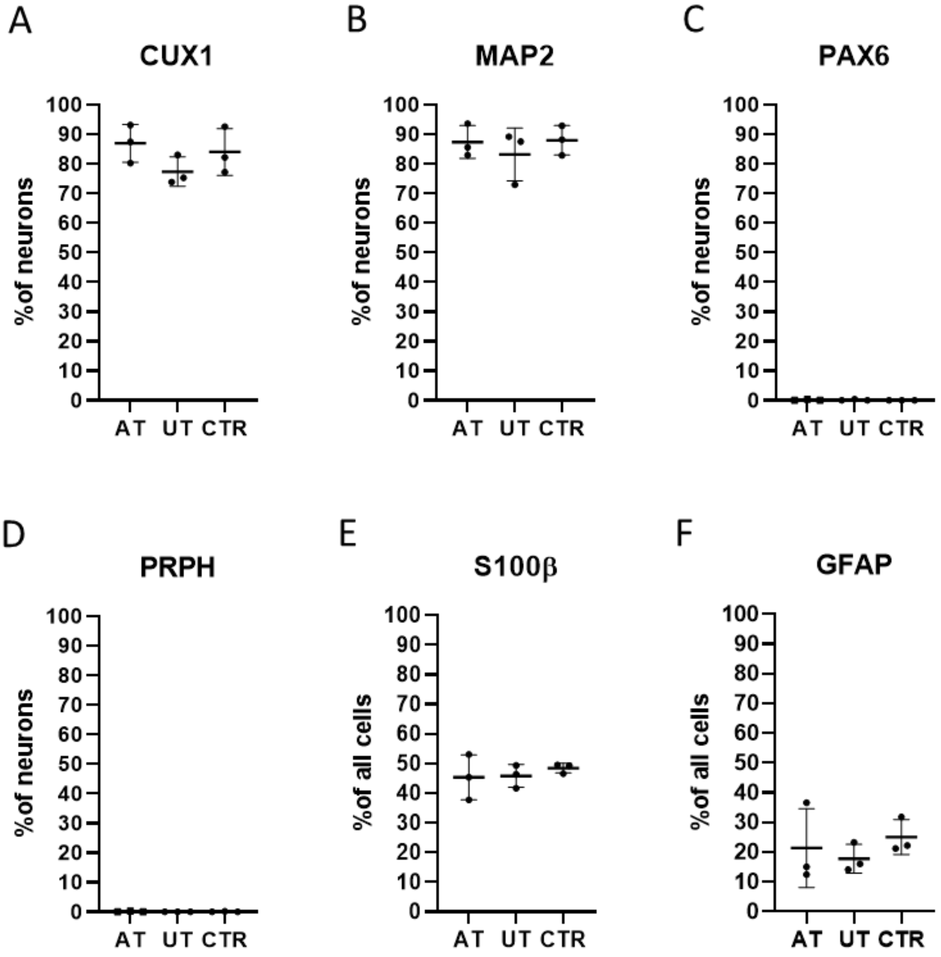

Supplemental Figure 6: Characterization of neuron-astrocyte co-cultures derived from monozygotic twins discordant for treatment-resistant schizophrenia. A-B. Neurons in cultures derived from affected twin (AT), unaffected twin (UT) and control cultures (CTR) were CUX1 and MAP2 positive. C-D. PAX6 and PRPH expressing cells were not detected in the cultures. E-F. Approximately 50% of the cells in all co-culture conditions expressed astroglial marker S100β and on average 20 % of the cells expressed GFAP. (n = 3 cell lines per group, data was collected from 2 independent experiments, each data point represents 2-4 replicate samples)

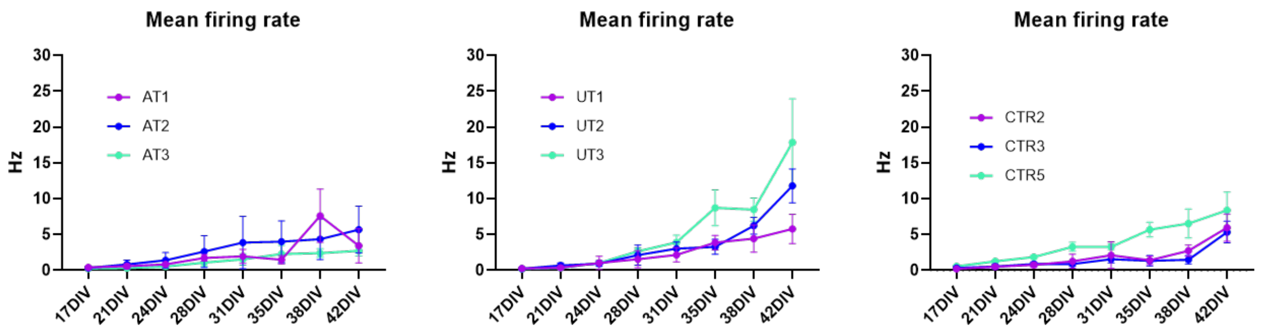

Supplemental Figure 7: MFR measured from neuron-astrocyte co-cultures from affected twins (AT), unaffected twins (UT) and control individuals (CTR).

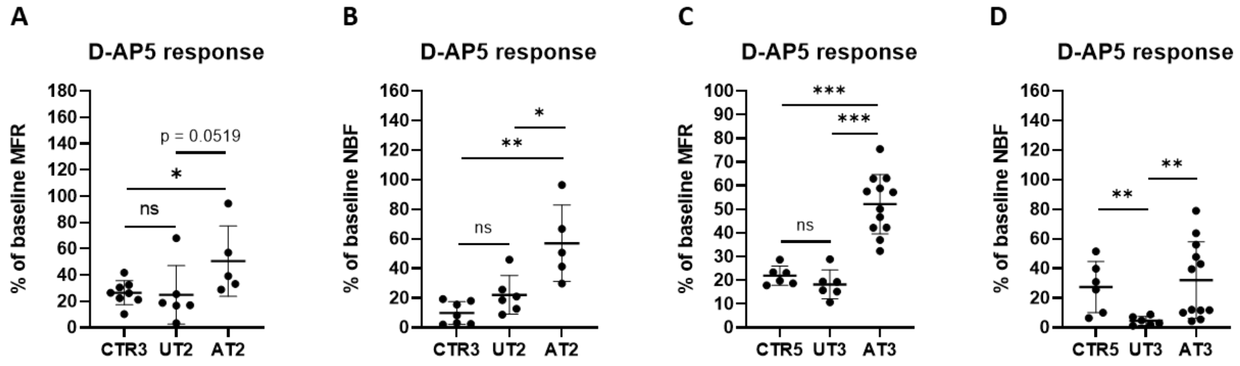

Supplemental Figure 8: D-AP5 responses in twin pairs 2 (A-B) and 3 (C-D). The UT cultures displayed a stronger response to D-AP5 in terms of MFR and NBF compared to AT cultures. (n = 5-12 samples per condition. The data was collected across 1-2 independent experiments)

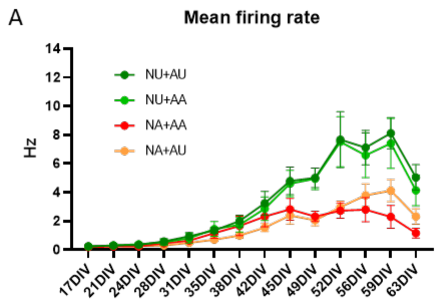

	NU+AU vs NU+AA	NU+AA vs NA+AA	NA+AA vs NA+AU	NU+AU vs AA+NU	NU+AU vs NA+AA	NU+AA vs NA+AU
17DIV	ns	ns	ns	ns	ns	ns
21DIV	ns	ns	ns	ns	ns	ns
24DIV	ns	p = 0.0411	ns	ns	ns	p = 0.0087
28DIV	ns	ns	p = 0.0411	p = 0.0022	ns	p = 0.0260
31DIV	ns	ns	ns	p = 0.0022	p = 0.0260	p = 0.0022
35DIV	ns	ns	p = 0.0260	p = 0.0022	ns	p = 0.0022
38DIV	ns	ns	p = 0.0260	p = 0.0022	ns	p = 0.0260
42DIV	ns	ns	p = 0.0411	p = 0.0022	ns	p = 0.0022
45DIV	ns	p = 0.0152	ns	p = 0.0022	p = 0.0043	p = 0.0043
49DIV	ns	p = 0.0022	ns	p = 0.0022	p = 0.0022	p = 0.0022
52DIV	ns	p = 0.0022	ns	p = 0.0022	p = 0.0022	p = 0.0022
56DIV	ns	p = 0.0022	ns	p = 0.0043	p = 0.0022	p = 0.0022
59DIV	ns	p = 0.0022	p = 0.0022	p = 0.0022	p = 0.0022	p = 0.0022
63DIV	ns	p = 0.0022	p = 0.0043	p = 0.0022	p = 0.0022	p = 0.0022

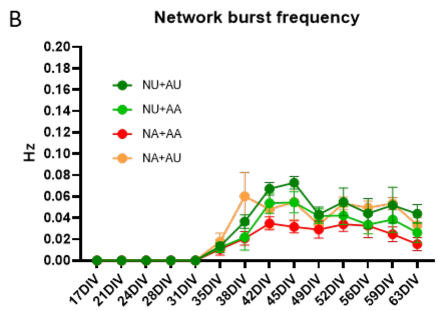

	NU+AU vs NU+AA	NU+AA vs NA+AA	NA+AA vs NA+AU	NU+AU vs NA+AU	NU+AU vs NA+AA	NU+AA vs NA+AU
17DIV	ns	ns	ns	ns	ns	ns
21DIV	ns	ns	ns	ns	ns	ns
24DIV	ns	ns	ns	ns	ns	ns
28DIV	ns	ns	ns	ns	ns	ns
31DIV	ns	ns	ns	ns	ns	ns
35DIV	ns	ns	ns	ns	ns	ns
38DIV	p = 0.0368	ns	p = 0.0238	ns	p = 0.0043	p = 0.0476
42DIV	p = 0.0238	p = 0.0043	ns	ns	p = 0.0022	ns
45DIV	p = 0.0043	p = 0.0022	p = 0.0022	ns	p = 0.0022	ns
49DIV	ns	p = 0.0281	ns	p = 0.0173	p = 0.0260	ns
52DIV	ns	ns	p = 0.0022	ns	p = 0.0022	p = 0.0238
56DIV	ns	ns	p = 0.0216	ns	p = 0.0455	p = 0.0043
59DIV	ns	ns	p = 0.0022	ns	p = 0.0043	p = 0.0238
63DIV	p = 0.0065	p = 0.0390	p = 0.0087	ns	p = 0.0022	ns

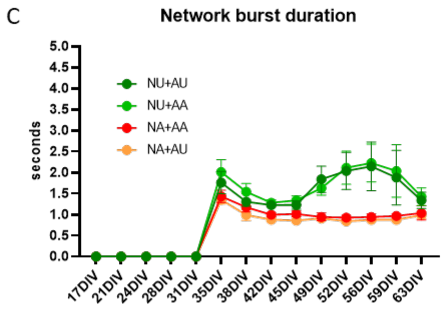

	NU+AU vs NU+AA	NU+AA vs NA+AA	NA+AA vs NA+AU	NU+AU vs NA+AU	NU+AU vs NA+AA	NU+AA vs NA+AU
17DIV	ns	ns	ns	ns	ns	ns
21DIV	ns	ns	ns	ns	ns	ns
24DIV	ns	ns	ns	ns	ns	ns
28DIV	ns	ns	ns	ns	ns	ns
31DIV	ns	ns	ns	ns	ns	ns
35DIV	ns	p = 0.0022	ns	p = 0.0043	p = 0.0260	p = 0.0022
38DIV	p = 0.0260	p = 0.0043	ns	p = 0.0238	p = 0.0411	p = 0.0238
42DIV	ns	p = 0.0022	p = 0.0152	p = 0.0022	p = 0.0022	p = 0.0022
45DIV	ns	p = 0.0022	p = 0.0022	p = 0.0022	p = 0.0043	p = 0.0022
49DIV	ns	p = 0.0022	ns	p = 0.0022	p = 0.0022	p = 0.0022
52DIV	ns	p = 0.0022	p = 0.0087	p = 0.0022	p = 0.0022	p = 0.0022
56DIV	ns	p = 0.0022	p = 0.0260	p = 0.0022	p = 0.0022	p = 0.0022
59DIV	ns	p = 0.0022	p = 0.0022	p = 0.0022	p = 0.0022	p = 0.0022
63DIV	ns	p = 0.0022	ns	p = 0.0043	p = 0.0260	p = 0.0022

Supplemental Figure 9: Development of MFR (A), NBF (B) and NBD (C) in co-cultures with neurons and astrocytes from UT (NU+AU), neurons from UT and astrocytes from AT (NU+AA), neurons and astrocytes from AT (NA+AA), and neurons from AT and astrocytes from UT (NA+AU). (n = 6 samples per condition, data was collected from 1 experiment. Mann-Whitney U test was used for the statistical comparisons)

References

1. Nehme R, Zuccaro E, Ghosh SD, Li C, Sherwood JL, Pietilainen O, *et al.* (2018): Combining NGN2 Programming with Developmental Patterning Generates Human Excitatory Neurons with NMDAR-Mediated Synaptic Transmission. *Cell Rep* 23. https://doi.org/10.1016/j.celrep.2018.04.066

2. Oksanen M, Petersen AJ, Naumenko N, Puttonen K, Lehtonen Š, Gubert Olivé M, *et al.* (2017): PSEN1 Mutant iPSC-Derived Model Reveals Severe Astrocyte Pathology in Alzheimer's Disease. *Stem Cell Reports* 9. https://doi.org/10.1016/j.stemcr.2017.10.016

3. Koskuvi M, Lehtonen Š, Trontti K, Keuters M, Wu YC, Koivisto H, *et al.* (2022): Contribution of astrocytes to familial risk and clinical manifestation of schizophrenia. *Glia* 70. https://doi.org/10.1002/glia.24131

4. Sahu MP, Nikkilä O, Lågas S, Kolehmainen S, Castrén E (2019): Culturing primary neurons from rat hippocampus and cortex. *Neuronal Signal* 3. https://doi.org/10.1042/ns20180207

5. Schildge S, Bohrer C, Beck K, Schachtrup C (2013): Isolation and culture of mouse cortical astrocytes. *J Vis Exp*. https://doi.org/10.3791/50079

6. Tiihonen J, Koskuvi M, Storvik M, Hyötyläinen I, Gao Y, Puttonen KA, *et al.* (2019): Sex-specific transcriptional and proteomic signatures in schizophrenia. *Nat Commun* 10. https://doi.org/10.1038/s41467-019-11797-3

7. Gordon A, Yoon SJ, Tran SS, Makinson CD, Park JY, Andersen J, *et al.* (2021): Long-term maturation of human cortical organoids matches key early postnatal transitions. *Nat Neurosci* 24. https://doi.org/10.1038/s41593-021-00802-y

8. Zhang WB, Ross PJ, Tu Y, Wang Y, Beggs S, Sengar AS, *et al.* (2016): Fyn Kinase regulates GluN2B

subunit-dominant NMDA receptors in human induced pluripotent stem cell-derived neurons. *Sci Rep* 6. https://doi.org/10.1038/srep23837

9. Muller PY, Janovjak H, Miserez AR, Dobbie Z (2002): Processing of gene expression data generated by quantitative real-time RT-PCR. *Biotechniques* 32.
